# Quantification and Evidence for Mechanically Metered Release of Pygidial Secretions in Formic Acid-Producing Carabid Beetles

**DOI:** 10.1673/031.010.1201

**Published:** 2010-03-02

**Authors:** Kipling W. Will, Aman S. Gill, Hyeunjoo Lee, Athula B. Attygalle

**Affiliations:** ^1^ Department of Environmental Science, Policy and Management, Division of Organisms and Environment, University of California, 137 Mulford Hall, Berkeley, CA 94720; ^2^ Department of Chemistry and Chemical Biology, Stevens Institute of Technology, Hoboken, New Jersey 07030

**Keywords:** pygidial glands, platynini, Sphodrini, chemical secretions, allomones, *Platynus brunneomarginatus*, *Platynusovipennis*, *Calathus ruficollis*

## Abstract

This study is the first to measure the quantity of pygidial gland secretions released defensively by carabid beetles (Coleoptera: Carabidae) and to accurately measure the relative quantity of formic acid contained in their pygidial gland reservoirs and spray emissions. Individuals of three typical formic acid producing species were induced to repeatedly spray, ultimately exhausting their chemical compound reserves. Beetles were subjected to faux attacks using forceps and weighed before and after each ejection of chemicals. *Platynus brunneomarginatus* (Mannerheim) (Platynini), *P. ovipennis* (Mannerheim) (Platynini) and *Calathus ruficollis* Dejean (Sphodrini), sprayed average quantities with standard error of 0.313 ± 0.172 mg, 0.337 ± 0.230 mg, and 0.197 ± 0.117 mg per spray event, respectively. The quantity an individual beetle released when induced to spray tended to decrease with each subsequent spray event. The quantity emitted in a single spray was correlated to the quantity held in the reservoirs at the time of spraying for beetles whose reserves are greater than the average amount emitted in a spray event. For beetles with a quantity less than the average amount sprayed in reserve there was no significant correlation. For beetles comparable in terms of size, physiological condition and gland reservoir fullness, the shape of the gland reservoirs and musculature determined that a similar effort at each spray event would mechanically meter out the release so that a greater amount was emitted when more was available in the reservoir. The average percentage of formic acid was established for these species as 34.2%, 73.5% and 34.1% for for *P. brunneomarginatus, P. ovipennis* and *C. ruficollis*, respectively. The average quantities of formic acid released by individuals of these species was less than two-thirds the amount shown to be lethal to ants in previously published experiments. However, the total quantity from multiple spray events from a single individual could aggregate to quantities at or above the lethal level, and lesser quantities are known to act as ant alarm pheromones. Using a model, one directed spray of the formic acid and hydrocarbon mix could spread to an area of 5–8 cm diameter and persisted for 9–22 seconds at a threshold level known to induce alarm behaviors in ants. These results show that carabid defensive secretions may act as a potent and relatively prolonged defense against ants or similar predators even at a sub-lethal dose.

## Introduction

The evolution and diversification of carabid beetles has been proposed to be tightly linked to changes in delivery, composition and potency of their defensive secretions (Erwin 1981; Will et al. 2000). Although the use of defensive allomones produced from the pygidial glands of adephagan beetles is well documented in terms of the various admixtures of compounds released (Dazzini-Valcurone and Pavan 1980; Dettner 1985; Will et al. 2000) and mechanisms of delivery of those compounds (Forsyth 1972; Moore 1979; Rossini et al. 1997; Eisner and Aneshansley 1982), there are no studies that measure the quantity released in a typical spray event.

Pygidial gland secretions are employed defensively, and in this context the actual amount released is important for several reasons, including that semiochemical concentration is a determinant in what behaviors are elicited. For example, knowledge of spray quantities may help clarify whether carabid defensive gland compounds, when applied to a given competitor or predator will tend to trigger escape responses, alarm behavior, death, or some alternative. The amount held in reserve after initial and successive chemical emissions, critical to further defense, may also be ecologically important. Thus, knowledge of how carabid beetles apportion defensive compounds will help establish the ecological context within which defensive chemistry can be best understood. This study is the first to measure the quantity of defensive gland secretions released defensively by carabid beetles.

In a study by Rossini et al. (1997) on *Galerita lecontei* several individual beetles were held in the laboratory for 3.5 months to ensure gland fullness and then sampled to determine the quantity of formic acid held in the pygidial glands. They determined that the formic acid content was approximately 80% of the estimated total gland contents, or about 4.56 ± 0.72 mg per beetle. They found that *G. lecontei* on average would spray 6.5 times before depletion and reasoned that a typical discharge of 0.7 mg of formic acid (∼0.8 mg total discharge) would be the average minimal quantity ejected. However, this average was based on a sample of only six individuals and a series of spray events from beetles with anywhere from replete to nearly empty reservoirs. Therefore these data cannot address changes in quantity over the series of spray events and is not conclusive regarding the amount ejected in a usual spray event.

Like *G. lecontei*, the beetles in this study, *Platynus brunneomarginatus* (Mannerheim) (Coleoptera: Carabidae: Platynini), *P. ovipennis* (Mannerheim) (Platynini) and *Calathus ruficollis* Dejean (Sphodrini), produce formic acid, a common compound in carabid beetles (Dazzini-Valcurone and Pavan 1980; Will et al. 2000) and other arthropods (Blum 1981). Formic acid is mixed with hydrocarbons in these taxa, creating an admixture known to be a potent toxin (Löfqvist 1977) and an alarm pheromone in ants (Löfqvist 1976). Hydrocarbons and other lipophilic compounds have frequently been associated with formic acid as surfactants (Eisner et al. 1961; Rossini et al. 1997; Schildknecht et al., 1968a) that increase the effectiveness of the primary irritant and are themselves potentially defensive (Peschke and Eisner 1987). When agitated, carabid beetles emit these defensive chemicals from their pygidial glands by oozing them onto the body (Moore 1979), crepitation of fluids directed via a flange of Coanda (Eisner and Aneshansley 1982), pulsatile crepitation (Aneshansley et al., 1969) or as is the case for all formic acid spraying beetles, like those in our study, by spraying an aimed mist or droplets (Moore 1979; Rossini et al. 1997).

## Materials and Methods

### Terms

Terminology for morphological structures follows Forsyth (1972). Herein a *spray event* refers to a single spray episode from a beetle, induced by a pinch on a meso- or meta-leg.

### The beetles

To determine the average quantity sprayed, specimens of two species of Platynini were used, *P. brunneomarginatus* (n = 31; 18 females and 13 males) and *P. ovipennis* (n = 17; 8 females and 9 males), and one species of Sphodrini, *C. ruflcollis* (n = 9; 4 females and 5 males). To determine the relative quantities of formic acid 18, eight and 30 individuals were used for *P. brunneomarginatus*, *P. ovipennis* and *C. ruficollis* respectively. Specimens were collected from October to December from four sites in California: 1. Concord, Contra Costa Co., 2. Berkeley, Alameda Co., 3. Big Creek, Monterey Co., and 4. Mt. Tamalpais, Marin Co. Beetles were transported to laboratory facilities at the University of California, Berkeley, and kept individually on damp sphagnum in a plastic, 5 cm diameter Petri dishes with snap-on covers. They were housed in a growth chamber with a day-length and temperature routine that simulated local conditions. Beetles were fed commercial dog food. To reduce the artifact of laboratory conditions beetles were collected with minimal handling to prevent premature release of defensive chemicals and processed within 10 days of collection.

### Dissection and gland preparation

Various internal parasites are known to attack adult carabid beetles (Thiele, 1977) and breeding conditions may have physiological and behavioral consequences that could impact this study. Therefore, breeding conditions and the macro-parasite load of individual beetles was determined by dissection after sampling. Individual beetles were killed by briefly freezing them. Abdomens were dissected under distilled water using a stereo-microscope. Testicular and accessory glands or ovary development was noted. Females with developed ova were further dissected and their spermatheca checked for sperm using a compound microscope. For individuals forced to spray to exhaustion, fluid volume in the gland reservoirs was measured and recorded.

For the determination of the relative amounts of formic acid, specimens were briefly frozen, their abdomens removed and pygidial glands excised. Each specimen's pair of gland reservoirs was retained if equivalent in size. One gland reservoir of each same-sized pair was sealed in a capillary tube for the quantitative analysis of formic acid. The other reservoir of the pair was placed on a microscopic slide and excess external water removed. For the accurate measurement of weight, multiple reservoirs of approximately the same size were accumulated on a single slide and the pooled series was used for weighing (Table 1). After weighing the full reservoirs, they were crushed and all fluid was removed by wicking and evaporation at room temperature. The weight of the remaining glandular tissue (cuticle and muscle) was measured. The weight difference between the full and empty glands provides the weight of the fluid in the glands (Table 1). The average for the series was used as an approximation of the individual weights.

**Table I:**
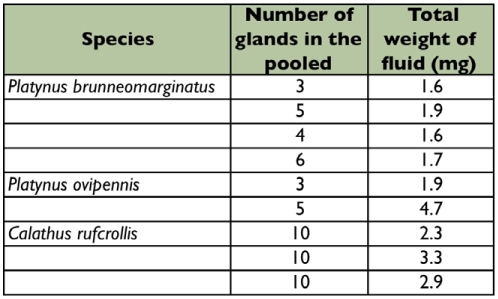
Total weight of pygidial gland fluid for *Platynus ovipennis*, *Platynus brunneomarginatus*, *and Calathus ruficollis.*

### Size measurements

As a standard measure of beetle body size, the area of the left elytron was used. Elytron length, from humeral angle to apex, and width along its widest point (approximate midpoint), were measured with a stereo-microscope equipped with an ocular reticule. In some studies the weight of an individual insect is reported, (e.g., Löfqvist 1977; Rossini et al. 1997), however, because weight varies depending on recent feeding, breeding condition, hydration, amount of stored fat body and quantity of stored defensive chemicals, weight is not a reliable metric for scaling size-related change in secretion output. The fixed size of the hard cuticle and relationship of elytra, which cover the abdomen to the capacity of the abdominal cavity, make elytron area (greatest length × greatest width) the preferred metric.

### Spray weight measurements

To obtain spray weight measurements, each beetle was affixed with an apparatus consisting of a piece of wire bent on one end into a hook, and on the other into a loop embedded in cheese wax, which was warmed and affixed to the beetle. To prevent defensive chemical discharge while the apparatus was being affixed and to promote wax adhesion, each beetle was placed in a -17 °C freezer for 4 min. The wire/wax apparatus was held over a flame to partially melt the wax, and was then attached to the beetle's elytra just after the beetle was removed from the freezer. The beetle was then suspended from a metal frame for 4 min to allow for a return to room temperature and normal locomotory behavior. Weights were taken using a Cahn Instruments C-31 microbalance, set at the 250 mg weight range. To simulate the humidity conditions of the damp sphagnum, a wet paper towel was kept in the chamber to maintain a high moisture level. Weights were considered reliable if a fluctuation of 0.002 mg or less was observed over 10 s. After an initial weight was taken, the beetle was held by the attached wire over filter paper soaked in an acid-indicating phenolphthalein-KOH solution. Typically the right meta-leg was pinched using forceps mounted with super glue onto a clothespin. A standardized pinch force was produced by allowing the clothespin spring to press the forceps closed onto the beetle's leg. The presence of defensive chemical secretion was indicated by smell and color change on the filter paper. The beetle was immediately re-weighed, and the difference between the second and first weight values was taken to be the mass of defensive chemicals secreted. Beetles were then returned to their respective Petri dishes to be re-tested the following day.

To induce beetles to spray to exhaustion, the procedure was as above, except beetles were pinched and re-weighed until no color change was seen on the reactive paper and no odor was detected. Re-weighing in the absence of color change or odor resulted in weights comparable to those obtained for negative controls (n=3), which were weighed, allowed to remain undisturbed for 30 s, and then reweighed.

Of the 31 individuals of *P. brunneomarginatus* a spray reaction was successfully induced in 23 for the first round (day one) and 29 in the second round (day two). Twenty individuals were successfully weighed in both rounds. Of the 17 individuals of *P. ovipennis* used, spray was induced in 16 for the first round and 17 in the second round. Of the nine individuals of *C. ruficollis* used, the spray reaction was induced in seven in the first round and nine in the second round

Seven (three female, four male) *P. brunneomarginatus* and three (two female, one male) *P. ovipennis* were induced to spray until exhaustion.

### Estimating amount contained in reservoirs at the time of a spray event

In the spray to exhaustion study the approximate quantity contained in the reservoirs was ascertained at the time of each spray event by summing weight differences from all spray events and then sequentially subtracting previous emission quantities (Table 2).

**Table 2:**
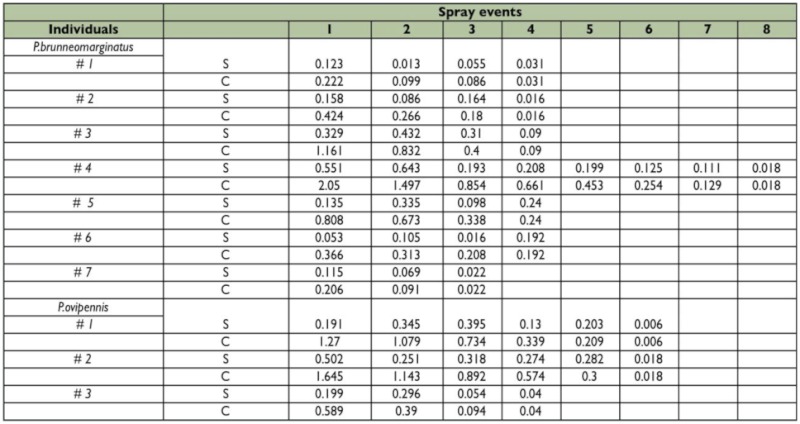
Spray to exhaustion study for each individual and spray event in milligrams. S- individual spray event, C- cumulative amount sprayed.

### Analytical chemistry methods

For GC-MS analysis, all reservoirs of the series in a capillary tube were carefully transferred into a vial (1 ml) filled with about 200 µl of dichloromethane. For a quantitative transfer, the empty capillary tube was thoroughly rinsed with dichloromethane and the rinsed fluid was carefully transferred into the vial containing the reservoirs. The reservoirs were crushed with a needle and the internal standard, 2 ml of pivalic acid solution (100 ppm) of dichloromethane was added to the crushed glands.

***Chemicals*.** Formic acid and pivalic acid were purchased from Aldrich Chemical Company, www.sigmaaldrich.com. Potassium hydrogen phthalate and sodium hydroxide were purchased from Fischer Scientific Company, www.fishersci.com.

***Instrumental*.** For the quantitative analysis of formic acid, one microliter aliquot of each sample was injected into a Shimadzu-QP5050 GC-MS (www.shimadzu.com) installed with a ZB-FFAP coated capillary column (30 m × 0.25 mm × 0.25 mm, Phenomenex, www.phenomenex.com). The oven temperature was initially held at 40 °C for 3 min, raised 6 °C/min to 250 °C, and held for 10 min. Helium was used as the carrier gas at a flow of 1.6 ml/min, and a split ratio of 12:1 on the Shimadzu instrument. The injections were made in splitless mode by an auto injector (Shimadzu, AOC-20i) on the Shimadzu-QP5050 GC-MS. Injection was repeated three times for each sample. Mass spectra were obtained under 70-eV electronionization with selected ion monitoring (m/z 41, 44, 45, 46, 57, 102) mode.

***Calibration curve*.** About 0.1 M NaOH solution was prepared and standardized with potassium hydrogen phthalate (110 °C for 2 h). The percentage of commercial formic acid was determined to be 98.58%) (w/w) by titration with the standardized NaOH solution. An internal standard, pivalic acid solution (100 ppm in dichloromethane), was accurately prepared. Stock solution (2500 ppm) of formic acid in dichloromethane was prepared and used to make 50, 100, 200, 300, 400, 500, and 600 ppm of standard solutions of formic acid. Each standard solution of formic acid (1 ml) and pivalic acid solution (1 ml, 100 ppm) was combined, and one microliter aliquot of the mixture was injected to a GC-MS. From the gas chromatogram, a calibration curve was plotted using the chromatographic area of formic acid/chromatographic area of pivalic acid versus the amount of formic acid/amount of pivalic acid, and the relative response factor was calculated

### Statistical methods

The two tailed t-test was used to determine if the two rounds of spray weights were significantly different, or were possible artifacts of our handling method, and if spray weights differed significantly between males and females of a species. Pearson correlation analysis was used to look for within- and between-species correlation for individual body size and quantity sprayed. Regression analysis was used to examine the relationship between amount contained in the pygidial gland reservoirs and amount sprayed. The final spray event — the spray that voids all remaining fluid in the reservoirs — was not included in this analysis as this spray event would always equal 100% of remaining fluid.

## Results

### Dissections

All individuals in this study were found to be free of macro-parasites. All individuals forced to spray to exhaustion were found to have empty gland reservoirs. Within species breeding condition was found to be comparable in individuals of the same sex. All *P. brunneomarginatus* males had a minute, undeveloped testis and scarcely any fluid in the accessory glands while females showed no sign of developing ova. All *P. ovipennis* males had a well-developed testis and significant quantities of fluid in the accessory glands (male *P. brunneomarginatus* and *P. ovipennis* have only one testis (Will et al., 2005)). Of the eight *P. ovipennis* females seven contained between seven and sixteen well developed eggs. No sperm was found in the spermathecae of any female *P. ovipennis*. All *C. ruficollis* males had well-developed testes and significant quantities of fluid in the accessory glands. Females had between two and four well developed eggs. No sperm was found in the spermathecae of any female *C. ruficollis*.

Nine to eleven different compounds were identified from analyses of whole-gland contents of the three species in this study (Table 3). Undecane, formic acid and doceyl acetate were the major compounds for *C. ruficollis.* Undecane, tridecanone, pentadecanone and formic acid are the major compounds present in *P. brunneomarginatus* and *P. ovipennis.*

**Table 3.**
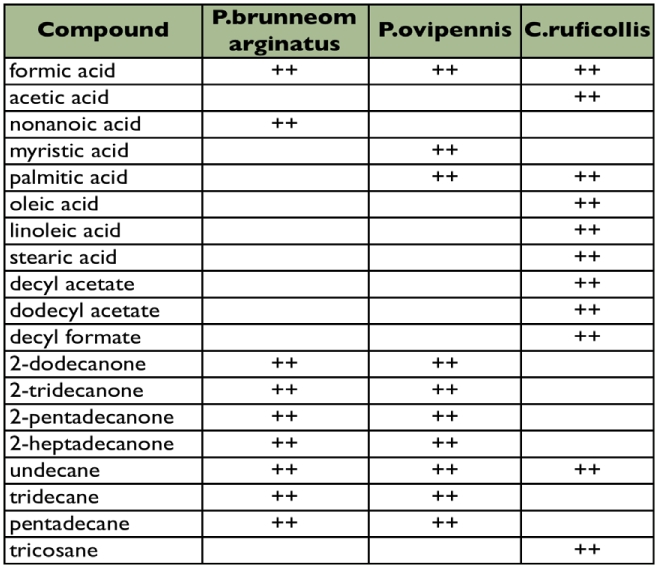
Volatile compounds characterized from carabid beetles in this study.

**Figure I:**
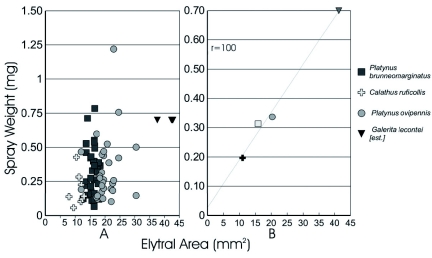
Relationship between elytral area and the weight of spray released in a spray event. A, plot of all species sampled in this study and *Galerita lecontei* (Rossini et al. 1997). B, Plot of species averages in this study and *G. lecontei* (Rossini et al. 1997). Elytral area for four typical individuals that were not part of the Rossini et al. study was measured for this graph. High quality figures are available online.

### Spray events

Difference in sequential weighing of negative controls was ∼ 0.01 mg. No significant difference was found between averages in round 1 and round 2 of spray weights within species (*P. brunneomarginatus* (P = 0.49), *P. ovipennis* (P = 0.97) and *C. ruficollis* (P = 0.45)). Within species weights were pooled from both rounds of spraying to give the overall average for each species.

No statistical difference was found between males and females for spray weights within species (*P. brunneomarginatus* (P = 0.55), *P. ovipennis* (P = 0.14) and *C. ruficollis* (P = 0.78)). Within species weights were pooled for both sexes.

Within species there was no correlation between size of an individual and the spray quantity emitted (Figure 1A). The Pearson correlation was calculated for spray quantity relative to elytral area for *P. brunneomarginatus* (r2 < 0.001), *P. ovipennis* (r2 < 0.032) and *C. ruficollis* (r2 < 0.001). For these three species average spray quantities were positively correlated to the average individual size for each species (r2 = 0.991, Figure 1B).

On average individuals of *P. brunneomarginatus* emit 0.313 ± 0.172 mg of pygidial secretions per spray event (median 0.318 mg; ∼ 0.370 µ 1); individuals of *P. ovipennis* emit 0.337 ± 0.229 mg (median 0.261mg; ∼ 0.398 µ1); and individuals of *C. ruficollis* emit 0.197 ± 0.117 mg (median 0.142 mg; ∼ 0.233 µl).

**Figure 2:**
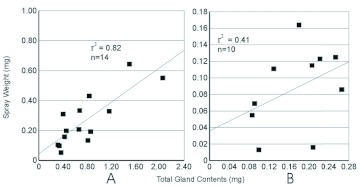
Quantities of chemicals held and sprayed by *Platynus brunneomarginatus*. A, weight of secretion contained in the gland reservoirs at each spray event. B, quantity released at each spray event.High quality figures are available online.

**Figure 3:**
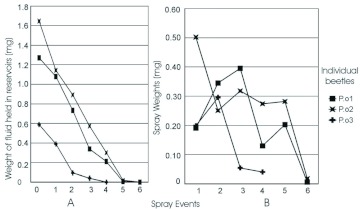
Quantities of chemicals held and sprayed by *Platynus ovipennis*. A, weight of secretion contained in the gland reservoirs at each spray event. B, quantity released at each spray event. High quality figures are available online.

In the spray to exhaustion study individuals of *P. brunneomarginatus* initially contained between 0.206 – 2.048 mg of secretion in their pygidial glands (Figure 2A) and *P. ovipennis* individuals ranged from 0.589 – 1.645 mg (Figure 3A). In *P. brunneomarginatus* three of seven individuals sprayed more in the second spray event, as did two of three *P. ovipennis.* Subsequent sprays tended to decrease in weight (Figure 2B, 3B).

**Figure 4:**
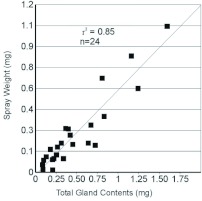
Plot and regression of all non-final spray events for spray to exhaustion study of *Platynus brunneomarginatus*. Weight of spray discharged on the Y axis, weight of fluid held in the gland reservoirs at the time of the spray event on the X axis. High quality figures are available online.

**Figure 5:**
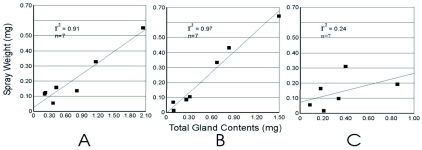
Plot and regression for first three spray events for spray to exhaustion study of *Platynus brunneomarginatus.* A, first spray events. B, second spray events. C, third spray events. Weight of spray discharged on the Y axis, weight of fluid held in the gland reservoirs at the time of the spray event on the X axis. High quality figures are available online.

**Figure 6:**
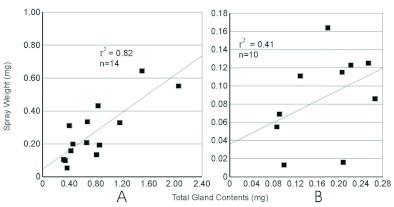
Plot and regression of spray events when reservoir contents are above and below average for *Platynus brunneomarginatus.* A, spray events for reservoirs at or above average discharge quantity. B, spray events for reservoirs below average discharge quantity excluding final discharge. Weight of spray discharged on the Y axis, weight of fluid held in the gland reservoirs at the time of the spray event on the X axis. High quality figures are available online.

Based on regression analysis, the amount emitted per spray event was positively correlated with the amount held in the beetle's reservoir at the time of spraying (r2 = 0.85, Figure 4). For *P. brunneomarginatus* the amount emitted in the first and second spray events was positively correlated with the amount held in the beetle's reservoir at the time of spraying (spray-1: r2 = 0.91, Figure 5A; spray-2: r2 = 0.97, Figure 5B). However, the third spray event was found to have a weak positive correlation (r2 = 0.24, Figure 5C). Regression analysis of only those spray events where individuals of *P. brunneomarginatus* had reserves at or greater than the average spray quantity for the species (≥ 0.31 mg) show a strong positive correlation (r2 = 0.82, Figure 6A), while spray events where beetles had reserves less than the average spray quantity (excluding final spray events) showed only a weak positive correlation (r2 = 0.41, Figure 6B).

**Table 4:**
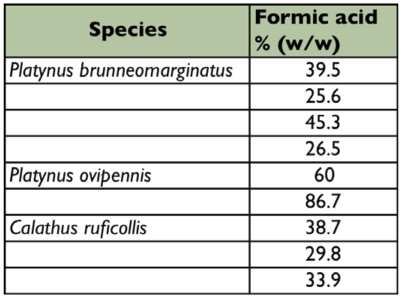
Percentage of formic acid (w/w) in the glandular fluid of carabid beetles in this study.

### Relative quantities of formic acid

On average the amount of formic acid contained in a single gland reservoir of *P. brunneomarginatus* is 34.2%, *P. ovipennis* 73.5% and *C. ruficollis* 34.2% (Table 4).

## Discussion

### Average spray events

Conspecific individuals showed no relationship between spray output and sex or body-size. There was no behavioral or physiological reason to expect a difference between the sexes of conspecifics. This is consistent with the expectation that males and females would encounter similar predators, requiring a similar defensive response. The average spray output did vary between species and was related to the average body size of the individuals sampled for that species. However, there was interspecific overlap (Figure 1A). Increases in spray distance as well as output relative to body size are expected as reservoir lumen area, quantity of muscle and abdomen size are known to be positively correlated (Forsyth, 1972). A relationship of size to output was not found within species, perhaps because the within species size-variation was quite small relative to the variation in secretion output. This is also possibly an artifact of sampling from a narrow part of species' ranges and a small sample of very similar individuals. Given the strong correlation for mean spray quantity and body size between species, it is likely that if the full range of sizes for each species was sampled a similar positive correlation may emerge. This does limit the extent to which we can suggest these results will be able to predict quantities of spray released by other species. However, it is likely that formic acid producing beetles of similar size and breeding condition will produce similar quantities of defensive chemicals.

### Spray quantity and lethality

The average individual spray event quantities in our study are well below the smallest quantity tested and found to be lethal to ants by (Löfqvist, 1977). In that study the smallest quantity of formic acid or hydrocarbons used was 0.5 µl (∼ 0.57 mg). The largest average single spray quantity we found was in individuals of *P. ovipennis*, which produce less than two-thirds the minimal amount used by Löfqvist. Löfqvist (1977) remarked that the quantities of formic acid used were probably similar to what one or a few ants could produce, but the hydrocarbons were perhaps as much as “10 times the total gland content in one ant.” However, Löfqvist considered that many ants may respond to a single threat with many individuals simultaneously spraying toxins. Carabid beetles are not known to be social beyond brooding and mate guarding behaviors (Anderson, 1984), and cooperative defense between individuals would not be expected. A single individual, however, can spray multiple times and does, typically, contain more than enough formic acid and hydrocarbon compounds to kill an individual ant. In the spray to exhaustion tests, five of the ten individuals emitted quantities equal to or up to about three times the 0.57 mg used by Lofqvist (1977) (Table 2). However, it seems unlikely that lethal toxicity is used or required against ants. An irritating or incapacitating effect and general disruption by eliciting an alarm signal is probably sufficient action. Variation in the ratio of the amount of defensive chemical emitted (Q) to the response threshold of the target (K) (Q/K of Bossert and Wilson (1963), see below) would be expected to yield different results ranging from death at a high Q/K to a more information rich message at lower Q/K.

### Alarm behavior

Several ant species have been shown to exhibit alarm behavior when exposed to formic acid and various hydrocarbons (Hölldobler and Wilson 1990; Löfqvist 1976) and the interaction of carabids is thought to be both ecologically and evolutionarily significant (e.g. Darlington 1971; Hawes et al. 2002; Reznikova and Dorosheva 2004; Liebherr and Krushelnycky 2006). Carabid formic acid-based chemical secretions would likely elicit a similar alarm effect in ants. Because of the frequent similarity of the compounds secreted this has even been considered a case of chemical mimicry (Blum 1980).

These data can be used to calculate a number of properties of a typical release and speculate on how this may affect ants. Three assumptions must be made to do this: 1) the diffusion constant (D) of formic acid in the sprayed mix is nearly the same as for formic acid alone (0.127cm2/sec (Gibson et al. 1997)), 2) the threshold value for an intense alarm behavior response to formic acid found for *Formica rufa* (K = 7×1015 molecules cm-3, Löfqvist 1976) is typical for ants, and 3) the formulae for diffusion from an instantaneous emission in still air can be applied as a reasonable model for an alarm semiochemical (Bossert and Wilson 1963). The maximum radius of the threshold sphere is then R_max_ = 0.527(Q/K)^1/3^ (Bossert and Wilson 1963, formula 1.4) where R is the radius, Q is the number of molecules released at the point of origin, and K is the behavioral threshold density. The time it takes to reach this maximum is TR_max_ = (0.0464/D)(Q/K)^2/3^ (Bossert and Wilson, 1963, formula 1.5), where D is the diffusion constant of formic acid. Based on this calculation, the average emission quantities found for single spray events for each of the three species in our study, and the relative quantity of formic found for these species in this study, it would be expected that an individual beetle would create a maximum of a 5–8 cm diameter hemisphere of threshold density for formic acid in 9–22 seconds (Table 5). Natural conditions in the field would be more complex than a simple hemispherical region of effect, but emissions based on this model do appear reasonable for beetles that would be expected to interact with ants and that are of a size-class where ants are a significant threat. The size of the region within the threshold density is sufficient to elicit behavioral responses from ants well away from contact with the beetle. It also persists long enough to allow a beetle to escape during the chemically induced confusion. Ants in immediate proximity to a beetle at the moment of spray release would of course be subject to a greater formic acid concentration than appears at R_max_.

**Table 5:**
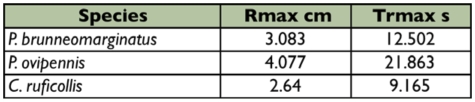
Calculated values for the maximum radius that would elicit an alarm response and time to reach the maximum. radius based on the threshold density for *Formica rufa* (Löfqvist, 1976) using the average spray weights and percentage of formic acid found in this study

### Spray to exhaustion

None of the species we sampled delivered a uniform quantity over a series of sequential spray events. The averages for spray quantities we found are much more accurate than the methods used to estimate spray output by Rossini et al. (1997) for *G. lecontei.* If their methods were used for our spray to exhaustion data, *P. brunneomarginatus* estimates would range from 0.056 to 0.290 mg for individuals and would have a 0.17 mg average over all events, while *P. ovipennis*, would be 0.15–0.27 mg, with an average of 0.22 mg overall. These are both well below our estimates for a single spray because the Rossini et al. (1997) method calculates the average including the terminal spray event (whatever remains in the reservoir) and other events with low reservoir volumes (less than average spray quantity available). The turgid condition of the gland reservoirs achieved in the laboratory after 3.5 months by *G. lecontei* in the Rossini et al. (1997) study is probably rarely found in nature where the glandular secretion might regularly be used. Therefore the average spray quantities found in our study probably represent a more realistic measure.

There are a number of conceivable ways that beetles could apportion their gland contents through sequential spray events, e.g., the quantities could have been random, constant for all events, increasing or decreasing. Beetles in this study exhibited a trend of decreasing emission quantities in sequential spray events (Figure 2–3). The quantity of fluid in the gland reservoir is apparently an important factor of, or at least highly correlated with, the quantity of fluid sprayed (Figure 2–4). One might be tempted to propose that beetles are actively assessing their defensive chemical reserve and ejecting a minimal amount that allows them to administer multiple sprays. To some extent this is possible; however this trend can be explained mechanistically without invoking a feed-back mechanism in the beetle.

### Action of the glands

The gland reservoirs are roughly ovoid in form, and have three layers of muscle, an outer layer running longitudinally and attached to the base of the efferent duct, an oblique middle layer attached to the dorsal lobe and an inner layer that attaches to the base of the collecting canal (Forsyth 1972). Given that any muscle has a maximum potential contraction length, if each muscle layer was recruited sequentially its contraction would be acting on a sequentially smaller volume as the reservoir is emptied, resulting in mechanically declining emissions. This can be roughly modeled by considering the gland to be an ovoid whose volume can be calculated as two elliptical paraboloids, base to base. The only way beetles could maintain or increase emission quantity with this system would be to exert an ever increasing amount of muscular effort. The increase in spray quantity shown between spray event 1 and event 2 in more than half the beetles (Figure 2B, 3B) is possibly due to a greater muscular effort or a bilateral rather than unilateral spray response. The methods we used cannot distinguish between use of both or only one of the contralateral pair.

The emission-reservoir volume relationship holds only when the reservoir volume is at or above the average spray weight quantity (Figure 6), as is normally the case during the first two sequential spray events (Figure 5). There is only a weak positive correlation between the reservoir content and spray weight once the reservoir content is below the average emission quantity. Reservoirs at this point are presumed to be nearly empty and the inner cuticle variously in-folded (Forsyth 1972), resulting in a very small inner volume.

### Chemical constituents

The admix of lipophilic and hydrophilic compounds present in the gland reservoirs of the three species in this study are consistent with what is known for other members of these genera (*P. assimilus, P. magnus, P. protensus*, *C. fucipes*, *C. melanocephalus* (Kanehisa and Kawazu 1985; Kanehisa and Murase 1977; Schildknecht 1970; Schildknecht et al. 1964; Schildknecht et al. 1968a; Schildknecht et al. 1968b)). However, these earlier studies only identified three or four major components in each species. We found a greater than twofold difference in the relative quantity of formic acid between *P. ovipennis* (73.5%) and the other two species (34%). This is notable, but not obviously explained by differences among these species. *Platynus ovipennis* is larger, more heavily sclerotized, flightless (as is *C. ruficollis*) and restricted to the wetter forests of the west coast of North America. *Calathus ruficollis* and *P. brunneomarginatus* are both very widespread species in the western United States. Both are found in relatively drier habitats. *Calathus ruficollis* is common in open grassy areas, while *P. brunneomarginatus* is generally near a source of water, e.g. permanent and vernal streams or springs. Too little is known of the specific ecological interactions, diet and behaviors of these beetles to know if any of these aspects are correlated with formic acid content.

### Limitations

A number of limitations of this study are important to point out. Sampling was specifically designed to limit the amount of within species variation due to physiological differences such as breeding condition and age. Samples were all collected from a very small number of sites in California and most of each species from a single site. The sample was of a comparable set of individuals in terms of probable age and physiological condition. This could mean the sample used is not necessarily representative of variation across the distribution of these beetles, and it may not reflect the potential effects of seasonal changes.

Our study also used a standard amount of stimulation to elicit the spray response. This means the full set of spray events are comparable measurements, probably representing the minimal stimulation needed to cause spray release. However, it does not address the question of possible variation in spray quantities that may be emitted under different levels of attack severity.

Our study establishes average quantities and variation of defensive chemicals in three carabid species. This finding provides a basis for further experimentation, using realistic quantities in bioassays, regarding the effect of the chemical constituents of carabid defensive compounds on ants and perhaps other likely predators, e.g. spiders, scorpions and other beetles.
